# Can treatment with the molecular adsorbent recirculation system be the solution for type-1 hepatorenal syndrome?

**DOI:** 10.1186/cc12368

**Published:** 2013-03-19

**Authors:** L Lavayssiere

**Affiliations:** 1 University Hospital, Toulouse, France

## Introduction

The aim of this retrospective study was to determine whether the molecular adsorbent recirculation system (MARS) can improve renal function in HSR1 patients.

## Methods

Thirty-two patients with chronic liver disease and HRS1 were treated by MARS sessions that were performed every other day. The endpoint was renal function improvement by 28 days after diagnosis of HRS1 that was defined as a serum creatinine level <133 μmol/l. Partial renal recovery was defined as a 10% decrease in baseline serum creatinine level.

## Results

The mean number of MARS sessions required by each patient was 3.5 ± 1.5. The median time between admission and the start of MARS therapy was 3 (0 to 15) days. Of the total patients, 13 (40%) had improved renal function. Among these, nine (28%) had complete renal recovery. Among the patients that survived, only 40% (6/15) had improved renal function. When MARS was started, 53% of patients had failed renal failure according to RIFLE criteria. Seven patients received a liver transplant after diagnosis of HRS. Of these, four had complete or partial recovery after transplantation (57%) versus nine of the 25 patients who did not undergo liver transplantation (36%), *P *= NS. The 28-day survival rate was 47%.

## Conclusion

MARS therapy improved renal function in only very few patients with HRS1. Even if liver transplantation is the best option, MARS could be used as a bridge until transplantation. Some authors argue that prolonged duration of dialysis (>1 month) before transplantation may result in chronic renal failure probably because there are two etiologies of AKI in end-stage liver disease: acute tubular necrosis (ATN) and real functional renal failure. ATN before liver transplantation has been identified as a risk factor for patients' mortality and chronic renal failure at 1 and 5 years post liver transplantation. Further prospective controlled studies including large number of patients are required.
